# Detection and Quantification of Calcium Ions in the Endoplasmic Reticulum and Cytoplasm of Cultured Cells Using Fluorescent Reporter Proteins and ImageJ Software

**DOI:** 10.21769/BioProtoc.4738

**Published:** 2023-08-20

**Authors:** Shunsuke Saito, Kazutoshi Mori

**Affiliations:** Department of Biophysics, Graduate School of Science, Kyoto University, Kyoto, Japan

**Keywords:** Calcium imaging, G-CEPIA1er, GCaMP6f, ImageJ, Endoplasmic reticulum, Cytoplasm, Cultured cells, Fluorescence microscopy

## Abstract

This protocol describes a method for detecting and quantifying calcium ions in the endoplasmic reticulum (ER) and cytoplasm of cultured cells using fluorescent reporter proteins and ImageJ software. Genetically engineered fluorescent reporter proteins, such as G-CEPIA1er and GCaMP6f, localize to intracellular regions of interest (i.e., ER and cytoplasm) and emit green fluorescence upon binding to calcium ions. In this way, the fluorescence brightness of cells transfected with expression vectors for these reporters reflects the calcium ion concentration in each intracellular region. Here, we describe procedures for observing cultured cells expressing these fluorescent reporters under a fluorescence microscope, analyzing the obtained image using the free image analysis software ImageJ (https://imagej.net/ij/index.html), and determining the average fluorescence brightness of multiple cells present in the image. The current method allows us to quickly and easily quantify calcium ions on an image containing multiple cells and to determine whether there are relative differences in intracellular calcium ion concentration among experiments with different conditions.

Key features

Detection and quantification of calcium ions in the ER and cytoplasm using fluorescent reporter proteins

Quick and easy verification of measurement results using ImageJ

Simultaneous comparison between various experimental conditions (drug treatment, mutants, etc.)

## Background

Calcium ions function as second messengers that mediate various signal transductions in cells (Berridge et al., 2000). Cytosolic calcium ions are actively taken up into the endoplasmic reticulum (ER) by sarcoplasmic calcium ATPase (SERCA), a calcium ion pump present in the ER membrane (Brini and Carafoli, 2009), to ensure that the calcium ion concentration in the ER is maintained at levels more than 5,000-fold higher than those in the cytoplasm (Laude ant Simpson, 2009). Calcium ions stored in the ER are released into the cytoplasm via the calcium ion channels inositol triphosphate receptor and ryanodine receptor as needed, and then rapidly recovered into the ER via SERCA. Such transient increase in cytosolic calcium ion concentration has been shown to be necessary for many biological phenomena, including muscle contraction and neurotransmission (Berridge et al., 2003).

At the same time, calcium ions are extremely important for maintaining protein homeostasis in the ER. The ER is where newly synthesized secretory and transmembrane proteins destined for the secretory pathway form the correct three-dimensional structure (Ellgaard and Helenius, 2003). BiP (immunoglobulin heavy chain–binding protein) is an Hsp70-type chaperone responsible for protein folding in the ER. For BiP to exert its activity normally, it is essential that a high concentration of calcium ions is maintained in the ER; BiP is a low-affinity and high-capacity calcium-binding protein (Preissler et al., 2020).

Based on these facts, disruption of calcium ion homeostasis in the ER not only impairs muscle and nerve function but also causes normal protein folding in the ER to be inhibited. As a result, the ER can enter a state termed ER stress, in which structurally abnormal proteins accumulate in the ER, which may in turn eventually lead to cell death. Over time, it was realized that such disruption of ER calcium ion homeostasis might actually be involved in aging-associated muscle dysfunction (Delrio-Lorenzo et al., 2020) and various neurodegenerative diseases (Schrank et al., 2020; Saito et al., 2022).

Traditional methods of detecting intracellular calcium ions have long used acetoxymethyl ester (AM)-conjugated versions of fluorescence-indicating chemicals such as Fura-2 AM, which mainly localizes in the cytoplasm, or Mag-Fura-2 AM, which localizes in the cytoplasm and ER. Fura-2 and Mag-Fura-2 themselves are composed of a calcium ion–chelating moiety and a fluorescent cluster moiety. The binding of calcium ions alters their conformation, resulting in changes in the efficiency with which they absorb excitation light and emit fluorescence. Importantly, conjugation of AM with these indicators inhibits their binding to calcium ions outside the cell and simultaneously confers membrane permeability on them (Tsien, 1981). Accordingly, these AM-conjugated indicators penetrate the plasma membrane, followed by removal of their AM parts by cellular esterase. This allows them to accumulate inside cells, bind to intracellular calcium ions, and emit fluorescence. As a result, calcium ion concentration in the cytoplasm can be easily measured using cells treated with Fura-2 AM.

In the case of Mag-Fura-2 AM, this is used by first determining the fluorescence intensity in cells treated with Mag-Fura-2 AM, and then by similarly determining intensity in these cells after treatment with digitonin or saponin, which permeabilize the plasma membrane but not the ER membrane. This second value represents calcium ion concentration in the ER; subtracting this from the first value reveals calcium ion concentration in the cytoplasm (Hofer and Machen, 1993).

In the current protocol, recently developed and genetically engineered fluorescent reporter proteins such as G-CEPIA1er (Suzuki et al., 2014) and GCaMP6f (Chen et al., 2013) are expressed in cultured cells separately by transfection. These are fusion proteins consisting of a circularly permutated fluorescent protein, a calmodulin domain, and the M13 fragment from myosin light chain kinase. Binding of calcium ions to the calmodulin domain causes a conformational change that results in the emission of fluorescence. Because of their optimized affinity for calcium ions, large dynamic range, and specific intracellular localization, fluorescence from G-CEPIA1er or GCaMP6f reports calcium ion dynamics in the ER and cytoplasm, respectively, in a single measurement. We explain how to quickly and easily test the results using ImageJ, a free analysis software. By applying this protocol, it is possible to detect and quantify ER and cytoplasmic calcium ions in a variety of plasmid-transfectable cells without complicated sample preparation procedures using different reagents.

## Materials and reagents

1. *Homo sapiens* neuroblastoma cell line SH-SY5Y (ATCC, catalog number: CRL-2266)
2. *Homo sapiens* colon colorectal carcinoma cell line HCT116 (ATCC, catalog number: CCL-247)
3. 24-well plate (for SH-SY5Y) (Corning, Falcon^®^, catalog number: 353047)
4. 6-well plate (for HCT116) (Corning, Falcon^®^, catalog number: 353046)
5. Dulbecco’s modified Eagle’s medium (DMEM) (Nacalai Tesque, catalog number: 08458-45)
6. Fetal bovine serum (FBS) (Thermo Fisher Scientific, Gibco^TM^, catalog number: 10270-106)
7. 100 U/mL penicillin and 100 μg/mL streptomycin (Nacalai Tesque, catalog number: 26253-84)
8. Opti-MEM (Thermo Fisher Scientific, Gibco^TM^, catalog number: 31985-070)
9. Polyethylenimine max (Polyscience, catalog number: 24765-100)
10. Lipofectamine^®^ LTX and Plus^TM^ Transfect (Thermo Fisher Scientific, Invitrogen^TM^, catalog number: 15338100)
11. pCMV G-CEPIA1er (Addgene, plasmid, catalog number: 58215)
12. pGP-CMV-GCaMP6f (Addgene, plasmid, catalog number: 40755)
13. pCMV-myc-wtSeipin (Satio et al., 2022)
14. pCMV-myc-ngSeipin (Satio et al., 2022)
15. 1 mM thapsigargin (Calbiochem, catalog number: T9033) dissolved in DMSO (stored at -20 °C)

## Equipment

1. Fluorescence stereomicroscope (Olympus IX-71-22TFL/PH)
2. Acquisition software (DP Controller 1.2.1.108)

## Software

1. ImageJ (https://imagej.net/ij/index.html)
2. Excel (Microsoft)

## Procedure

- **Detection and quantification of calcium ions in the ER**
  1. Preparation of cultured cells expressing reporter protein
    - Culture SH-SY5Y cells in DMEM with glucose (4.5 g/L) supplemented with 10% FBS and antibiotics (100 U/mL penicillin and 100 μg/mL streptomycin) in an incubator at 37 °C and a 5% CO_2_ atmosphere.
    - Transfect SH-SY5Y cells (3.0 × 10^5^ cells, approximately 80% confluent on a 24-well plate) with 200 ng of pCMV G-CEPIA1er in 63 μL of Opti-MEM, 3.2 μL of Lipofectamine^®︎ ^LTX, and 0.6 μL of Plus^TM^ Transfect.
  2. Detection and acquisition of images of cells expressing reporter protein
    - Twenty-eight hours after transfection, observe the cells under an inverted fluorescence microscope (e.g., Olympus IX-71-22TFL/PH) with an appropriate exposure time. If phenol red contained in DMEM causes a strong background signal, the volume of DMEM should be accordingly reduced. If a sufficient number of cells is present on the dish (approximately 95% confluent) and transfection efficiency is sufficient (approximately 15%), images should be taken using a 20× objective lens. This will produce images that contain approximately 100 fluorescent cells per image (Figure 1).
    **Figure 1.**
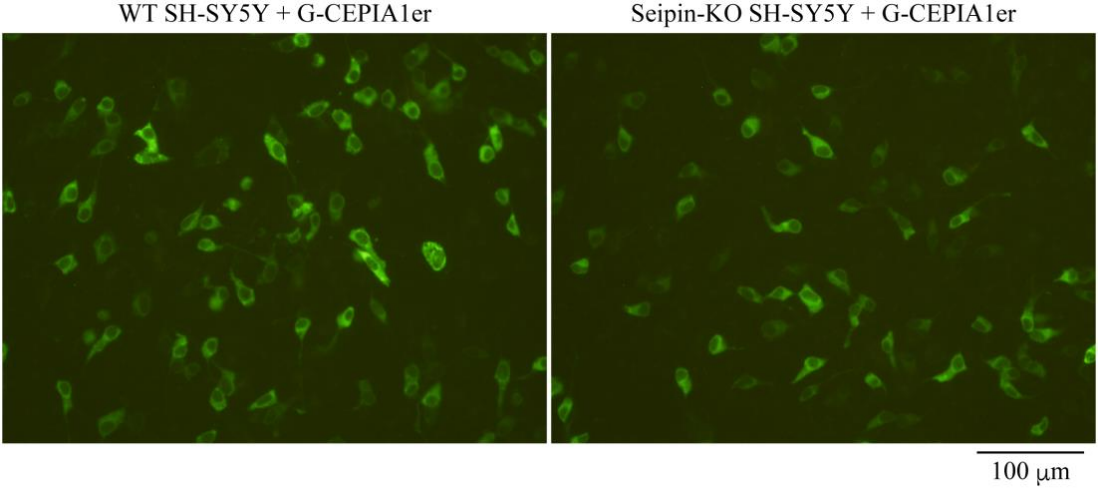
Fluorescence images of wild-type (WT) and *Seipin*-knockout (KO) SH-SY5Y cells expressing G-CEPIA1er. WT or *Seipin*-KO SH-SY5Y cells seeded on 24-well plates were transfected with 200 ng of pCMV G-CEPIA1er and observed for fluorescence 28 h later. Scale bar, 100 μm.
  3. Quantification of fluorescence by ImageJ
    - Export the obtained images in tif or another appropriate file format and open them in ImageJ (Figure 2A).
    - Select Image > Color > Split Channels to split each image into Red, Green, and Blue channels. Of these, use only the Green-channel image for quantification and close the others (Figure 2B).
    - Select Process > Subtract Background to perform background subtraction, with Rolling Ball Radius = 50.0 pixels (Figure 2C and 2D).
    - Select Adjust > Threshold to set the threshold such that the quantitative range is limited only to the area where cells are present (Figure 2E and 2F).
    - Select Analysis > Set Measurement and check the “Mean grey Value” and “Limit to threshold” checkboxes (Figure 2G).
    - Select Analysis > Measure to obtain the quantification results (Figure 2H).
  4. Statistical analysis in Microsoft Excel (Figure 3)
    - Paste the results from Figure 2H (A1:B17 and D1:E16).
    - Calculate the average of the results of the group that should be used as the reference (B21).
    - Divide each result by the average of the reference group (A25:B41 and D25:E40).
    - Calculate the average (H26 and I26) and standard deviation (H27 and I27) of the divided result of each group.
    - Use the F-test to determine if the variances are equal between the divided results of the two groups being compared (L26) and use Student’s *t*-test to determine if there is a significant difference between the divided results of the two groups (L27).
    Figure 4 shows detection and quantification of calcium ions in the ER of wild-type and *Seipin*-knockout SH-SY5Y cells using this protocol.
    **Figure 2.**
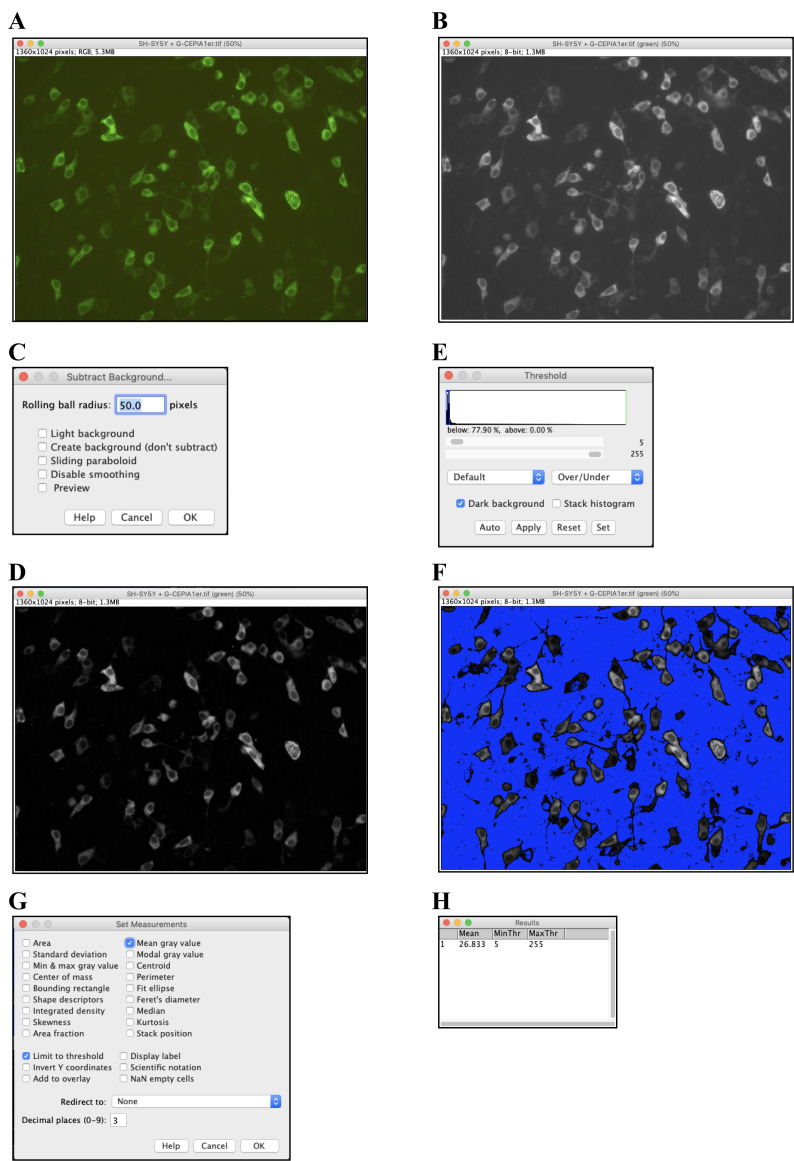
Procedures for analyzing fluorescence images using ImageJ. A. The tif file of the fluorescence image from Figure 1A opened in ImageJ. B. By selecting Image > Color > Split Channels and splitting each image into Red, Green, and Blue channels, a Green-channel image was obtained. C. and D. By selecting Process > Subtract Background, background subtraction was performed with Rolling Ball Radius = 50.0 pixels (C). This produced a background-subtracted version of the image (D). E, F. By selecting Adjust > Threshold, the threshold was set such that the quantitative range was limited to areas where cells were present (E). With this step, cell-free areas (blue) were marked and excluded from the analysis area (F). G. After selecting Analysis > Set Measurement, checkboxes for “Mean grey Value” and “Limit to threshold” were checked. H. Selecting Analysis > Measure provided the quantification results (mean = 26.833).
    **Figure 3.**
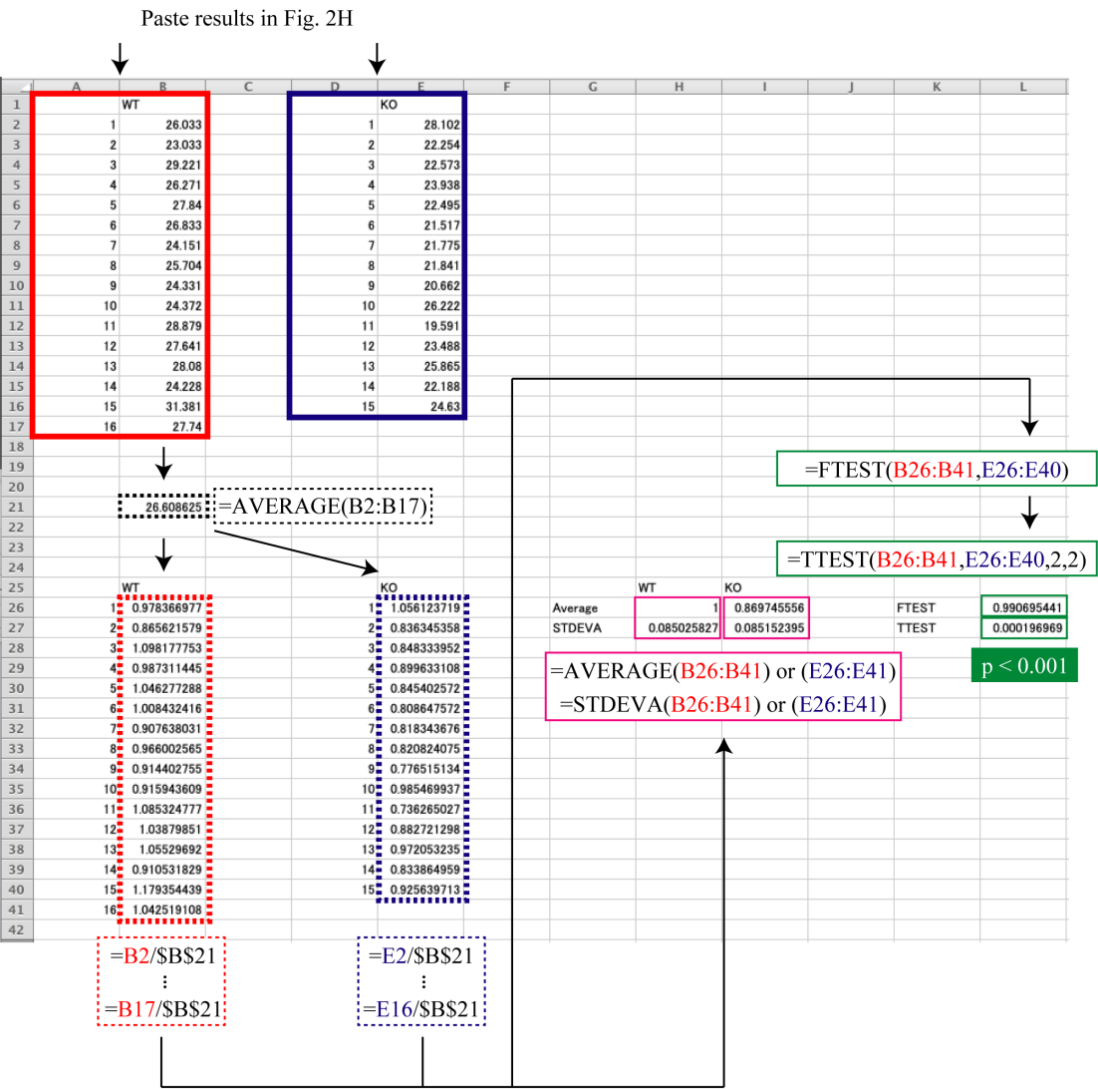
An example of the statistical data analysis. The results from each group (acquired following the procedure described in Figure 2) are pasted in A1:B17 and D1:E16. The average of results of the reference group (A1:B17 in this example) is calculated in B21. The result of dividing each result by the average of the reference group (B21) is shown in A25:B41 and D25:E40. The results of calculating the average and standard deviation for the divided result of each group are shown in H26 and I26, and H27 and I27, respectively. The results of the F-test to determine if the variances were equal between the divided results of the two groups being compared are shown in L26, and the result of Student’s *t*-test to determine if there was a significant difference between the divided results of the two groups is shown in L27.
    **Figure 4.**
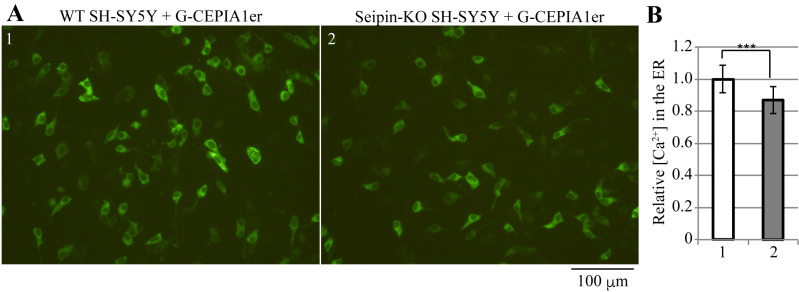
Calcium ion concentrations in the endoplasmic reticulum (ER) of wild-type WT and *Seipin*-knockout (KO) SH-SY5Y cells. A. WT (1) or *Seipin*-KO (2) SH-SY5Y cells seeded on 24-well plates were transfected with 200 ng of pCMV G-CEPIA1er and observed for fluorescence 28 h later. Scale bar, 100 μm. B. For both cell types, 5–6 images were taken per sample, and the experimental results for three samples (15–16 images in total) were analyzed following the procedures described in Figure 2 and statistically processed following the procedures described in Figure 3. The results are expressed as relative values, with the mean of measurements of WT SH-SY5Y cells set as 1, along with the standard deviation and presence of significant differences (Student’s *t*-test, ***: p < 0.001). The results show that *Seipin*-KO SH-SY5Y cells have a significantly lower calcium ion concentration in the ER than WT cells (Saito et al., 2022).
- **Detection and quantification of calcium ions in the cytoplasm**
  1. Preparation of cultured cells expressing reporter protein
    - Culture HCT116 cells in DMEM (glucose 4.5 g/L) supplemented with 10% FBS and antibiotics (100 U/mL penicillin and 100 μg/mL streptomycin) in an incubator at 37 °C and a 5% CO_2_ atmosphere.
    - Co-transfect HCT116 cells (8.0 × 10^5^ cells, approximately 60% confluent on 6-well plates) with 1 μg of pGP-CMV-GCaMP6f and 100 ng of pCMV-myc-wtSeipin or pCMV-myc-ngSeipin in 300 μL of Opti-MEM and 10 μL of polyethylenimine max solution (1 mg/mL in MilliQ water). HCT116 cells proliferate faster than SH-SY5Y cells; accordingly, they should be less confluent during transfection than SH-SY5Y cells.
  2. Detection and acquisition of images of cells expressing reporter protein
    - Twenty-eight hours after transfection, observe the cells under an inverted fluorescence microscope (e.g., Olympus IX-71-22TFL/PH) with an appropriate exposure time. If phenol red contained in DMEM causes a strong background signal, the volume of DMEM should be accordingly reduced. If a sufficient number of cells is present on the dish (approximately 95%) and transfection efficiency is sufficient (approximately 40%–50%), images should be taken using a 20× objective lens. This will produce images that contain approximately 150–200 fluorescent cells per image (Figure 5A).
    - Add 1 µM thapsigargin, an inhibitor of SERCA (Lytton et al., 1991), to the cells to evoke the leakage of calcium ions stored in the ER to the cytoplasm.
  3. Quantification of fluorescence by ImageJ (Figure 5BC)
    Quantify the images according to the procedures described in step A3.
  4. Statistical analysis on Microsoft Excel (Figure 5BC)
    Perform statistical analysis according to the procedures described in step A4.
    **Figure 5.**
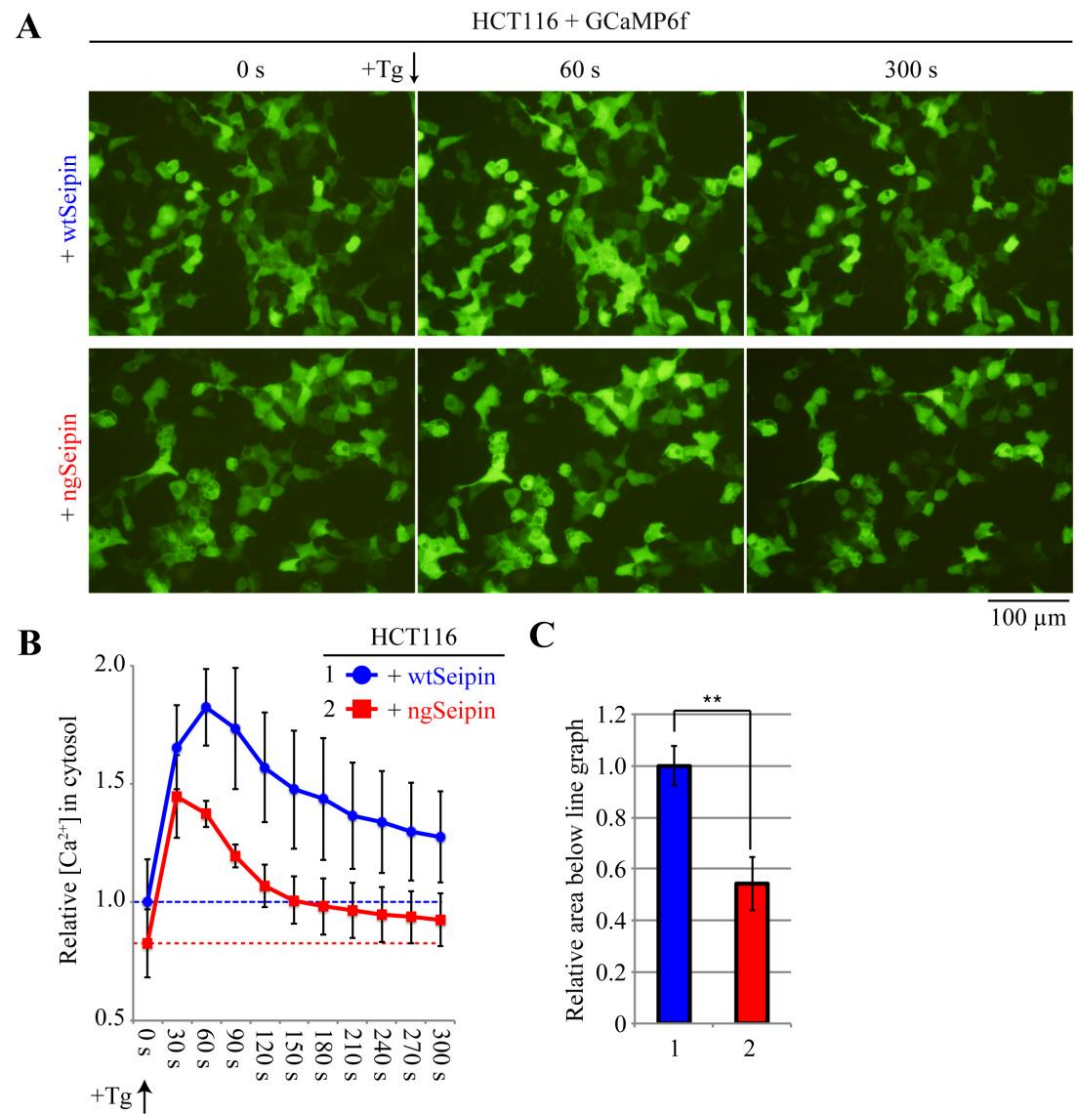
Calcium ion concentrations in the cytoplasm of HCT116 cells expressing wtSeipin or ngSeipin before or after treatment with thapsigargin. A. HCT116 cells seeded on 6-well plates were co-transfected with 1 μg of pGP-CMV-GCaMP6f and 100 ng of pCMV-myc-wtSeipin or pCMV-myc-ngSeipin. Twenty-eight hours later, cells were treated with or without 1 μM thapsigargin (Tg), which inhibits the sarcoplasmic calcium ATPase (SERCA) (Lytton et al., 1991) and thereby evokes calcium ion leakage from the endoplasmic reticulum (ER) to cytoplasm. 0, 60, and 300 s later, cells were observed for fluorescence. Scale bar, 100 μm. B. For both cell types, images were taken every 30 s after treatment with Tg, and results from three experiments were analyzed according to the procedures described in Figure 2 and statistically processed according to the procedures described in Figure 3. The results are expressed as relative values, with the mean of measurements of cells expressing myc-wtSeipin in 0 s set as 1, along with the standard deviation. C. To estimate the total amount of calcium ions released from the ER to cytoplasm by Tg treatment, the area below the line graph until the broken line (fluorescence intensity in 0 s) in B was calculated and expressed as relative values, with the mean of measurements of cells expressing myc-wtSeipin set as 1, along with the standard deviation and presence of significant differences (Student’s *t*-test, **: p < 0.01). The results show that ngSeipin decreases the calcium ions stored in the ER (Saito et al., 2022).

## Notes

Although this experimental method can be applied to various types of cultured cells, the method for introducing plasmids to express fluorescent reporter proteins (e.g., scale of cell culture, reagent types, volume of plasmid to be introduced, length of incubation time after transfection) needs to be optimized for each cell type. If the chemical gene transfer techniques using Polyethylenimine Max or Lipofectamine^® ^LTX are not effective, the use of viral vectors or electroporation should be considered.
